# Supplementary material for: Identification of Freezing-Responsive microRNAs and Their Targets in Chinese Jujube by Small RNA and Degradome Sequencing
Source: Int J Mol Sci. 2026 Sep 4;27(17):7912. doi: 10.3390/ijms27177912 (PMC13566890; doi:10.3390/ijms27177912)
Supplement: Supplementary file 1 [file ijms-27-07912-s001.zip › Supplementary Material S2/novel_miR_163.pdf]

Provisional ID	:	novel_miR_163
Score total	:	91
Score for star read(s)	:	3.9
Score for read counts	:	84.2
Score for mfe	:	1.3
Score for randfold	:	1.6
Score for cons. seed	:	
Total read count	:	177
Mature read count	:	147
Loop read count	:	0
Star read count	:	30

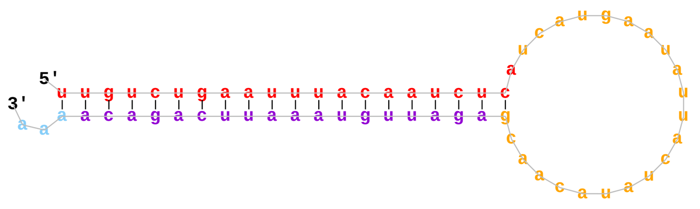

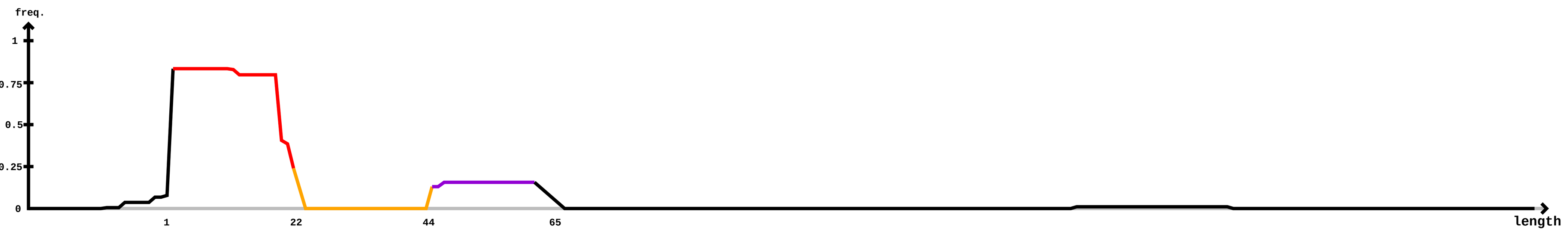

Mature **Star**

```
5'- ugaccuuuguaagaauugcauuuugucugaauuuacaaucucaucaugaauauuacuauacaacgagauuguaaauucagacaaaauucaaugucuugcaaauuaauucuccacuagcaacuacuaggagaagagccagcguaacucuauaaauagagcuugagcuaaauaaaauagacgcgugaaaauaaugcuggccgucacuuucgacuuuugguaggacaaauuuuaugaaaacacgcgucaugaa -3'  obs
    ugaccuuuguaagaauugcauuuugucugaauuuacaaucucaucaugaauauuacuauacaacgagauuguaaauucagacaaaauucaaugucuugcaaauuaauucuccacuagcaacuacuaggagaagagccagcguaacucuauaaauagagcuugagcuaaauaaaauagacgcgugaaaauaaugcuggccgucacuuucgacuuuugguaggacaaauuuuaugaaaacacgcgucaugaa       exp
    .....(((((((((((((.(((((((((((((((((((((((.....................)))))))))))))))))))))))).)))).)))))))))....((((((..(((......))))))))).....(((.((((((((....))))).))).)))..........((((((((...((((((((.((((((......)))....))).)).))....)))).....)))))))).....
```

alignment	reads	mm	sample
...........agaUuugcauuuugucugaau	1	1	LT-2
..............Uuugcauuuugucugaauu	6	1	LCK-3
...................auuuugucugaauuuacaauc	6	0	LCK-3
.....................uuugucugaauuuacaaucuc	2	0	LCK-3
......................uugucugaauuuacaauc	21	0	LCK-1
......................uugucugaauuuacaauc	3	0	LT-1
......................uugucugaauuuacaauc	7	0	LT-3
......................uugucugaauuuacaCuc	1	1	LCK-3
......................uugucugaauuuacaauc	3	0	LT-2
......................uugucugaauuuacaauc	7	0	LCK-3
......................uugucugaauuuacaauc	27	0	LCK-2
......................uugucugaauuuacaaucu	1	0	LT-3
......................uugucugaauuuacaauUu	2	1	LT-3
......................uugucugaauuuacaaucu	1	0	LT-1
......................uugucugaauuuacaaucuc	7	0	LT-1
......................uugucugaauuuacaauUuc	4	1	LT-2
......................uugucugaauuuacaaucuU	1	1	LT-1
......................uugucugaauuuacaaucuc	2	0	LT-2
......................uugucugCauuuacaaucuc	2	1	LT-1
......................uugucugaauuuacaaucuc	2	0	JCK-2
......................uugucugaauuuacaaucuc	8	0	LCK-3
......................uugucugaauuuacaaucucU	17	1	LCK-2
......................uugucugaauuuacaaucucU	29	1	LCK-1
....agauuguaaauucagaca	6	0	LT-2
....agauuguaaauucagaca	13	0	LCK-3
....agauuguaaauucagaca	1	0	JCK-3
....agauuguaaauucagacaaa	1	0	LT-1
....agauuguaaauucagacaaaa	3	0	LT-2
....agauuguaaauucagacaaaa	1	0	LT-1
....auuguaaauucagacaaaau	5	0	LCK-3
aauagacgcgugaaaauaaugcuggc	2	0	JCK-2
